# Impact of residual stress on coronary plaque stress/strain calculations using optical coherence tomography image-based multi-layer models

**DOI:** 10.3389/fcvm.2024.1395257

**Published:** 2024-04-25

**Authors:** Mengde Huang, Akiko Maehara, Dalin Tang, Jian Zhu, Liang Wang, Rui Lv, Yanwen Zhu, Xiaoguo Zhang, Chen Zhao, Haibo Jia, Gary S. Mintz

**Affiliations:** ^1^ School of Biological Science and Medical Engineering, Southeast University, Nanjing, China; ^2^ The Cardiovascular Research Foundation, Columbia University, New York, NY, United States; ^3^ Mathematical Sciences Department, Worcester Polytechnic Institute, Worcester, MA, United States; ^4^ Department of Cardiology, Zhongda Hospital, Southeast University, Nanjing, China; ^5^ Department of Cardiac Surgery, Shandong Second Provincial General Hospital, Jinan, China; ^6^ Department of Cardiology, The Second Affiliated Hospital of Harbin Medical University, Harbin, China

**Keywords:** residual stress, opening angle, coronary plaque, multi-layer artery model, plaque stress

## Abstract

**Introduction:**

Mechanical stress and strain conditions play an important role in atherosclerosis plaque progression, remodeling and potential rupture and may be used in plaque vulnerability assessment for better clinical diagnosis and treatment decisions. Single layer plaque models without residual stress have been widely used due to unavailability of multi-layer image segmentation method and residual stress data. However, vessel layered structure and residual stress have large impact on stress/strain calculations and should be included in the models.

**Methods:**

In this study, intravascular optical coherence tomography (OCT) data of coronary plaques from 10 patients were acquired and segmented to obtain the three-layer vessel structure using an in-house automatic segmentation algorithm. Multi- and single-layer 3D thin-slice biomechanical plaque models with and without residual stress were constructed to assess the impact of residual stress on stress/strain calculations.

**Results:**

Our results showed that residual stress led to a more uniform stress distribution across the vessel wall, with considerable plaque stress/strain decrease on inner wall and increase on vessel out-wall. Multi-layer model with residual stress inclusion reduced inner wall maximum and mean plaque stresses by 38.57% and 59.70%, and increased out-wall maximum and mean plaque stresses by 572.84% and 432.03%.

**Conclusion:**

These findings demonstrated the importance of multi-layer modeling with residual stress for more accurate plaque stress/strain calculations, which will have great impact in plaque cap stress calculation and plaque rupture risk assessment. Further large-scale studies are needed to validate our findings.

## 1 Introduction

Mechanical stress/strain conditions play an important role in atherosclerosis plaque progression, remodeling and potential rupture (1–6). Accurate models serve as the basis for plaque stress/strain calculations and the subsequent prediction of plaque progression and rupture. While it is well known that arteries have three-layer structure and residual stress (7, 8), single-layer models without residual stress were used in most current publications due to lack of available multi-layer image and residual stress data. With unprecedented optical coherence tomography (OCT) resolution (5–15 μm), we introduced a multi-layer OCT image segmentation method and multi-layer plaque models recently to demonstrate the impact of multi-layer structure on plaque stress/strain conditions (9, 10). In this paper, OCT-based multi-layer coronary plaque models with and without residual stress inclusion were constructed and compared with single-layer models to investigate the influence of model residual stress inclusion on plaque stress/strain calculations.

Residual stress, defined as the stress remaining in an *ex vivo* vessel ring under no-load condition, was initially observed by Fung and his colleagues (7, 11). When the vessel ring is cut open radially, the inherent residual stress causes the ring to spring open, forming a sector with a specific opening angle. Following this discovery, Holzapfel et al. revealed a diverse range of opening angles across different layers by experiments, with the media layer's angle may exceeding 180 degrees (12, 13).

Residual stress inclusion in vessel models may have considerable impact on vessel stress and strain distributions. Vito and Delfino et al. reported that incorporating residual stress led to a more uniform circumferential stress distribution in arterial models (8, 14). Ohayon et al. found that peak strain in coronary artery models is significantly overestimated when residual stress is not considered (15). Wang et al. demonstrated that residual stress led to reduced lumen and increased out-wall stress (16). Pierce et al. observed the impact of residual stress on the deformation and stress distribution within arterial tissue in abdominal aortic aneurysms models (17).

In this paper, patient-specific multi-layer and single thin-slice models with and without residual stress inclusion for coronary plaques were constructed using a three-step modelling procedure based on segmented OCT image data. Stress/strain results from vessel inner- and out-wall were extracted for comparison analysis. Patient variations of model differences were also observed.

## 2 Materials and methods

### 2.1 Data acquisition and multi-layer segmentation

Intravascular optical coherence tomography (OCT) coronary plaque data sets from 10 patients (8 male; 2 female) were used in this study. Of the 10 patients, 4 existing de-identified OCT data sets were obtained from Cardiovascular Research Foundation (CRF, New York, New York); 4 OCT data sets were acquired from Southeast University Affiliated Zhongda Hospital using protocol approved by Southeast University Zhongda Hospital Institutional Review Board (approval code 2019ZDKYSB046) with informed consent obtained. 2 existing de-identified OCT data sets were obtained from The Second Affiliated Hospital of Harbin Medical University. OCT images were acquired with ILUMIEN OPTIS System and Dragonfly JP Imaging Catheter (St. Jude Medical, Westford, Massachusetts). Patient demographic information is given in Table 1.

**Table 1:** Patient demographic data and clinical information.

Patient	Age	Sex	Vessel segment	BP(mmHg)	Comorbidities
P1	70	M	RCA	155/84	HT
P2	66	M	LCX	150/89	DM
P3	61	M	LCX	128/78	HT DM HL
P4	72	M	LCX	143/80	HT DM HL
P5	56	M	LAD	115/64	HT HL
P6	55	M	LAD	130/90	HT
P7	65	M	LAD	124/84	N/A
P8	50	F	LAD	175/92	HT HL
P9	43	M	LAD	132/90	HL
P10	59	F	LAD	121/71	HT HL

BP, blood pressure; RCA, right coronary artery; LCX, left circumflex artery; LAD, left anterior descending artery; HT, hypertension; DM, diabetes mellitus; HL, hyperlipoproteinemia.

**Figure 1:**
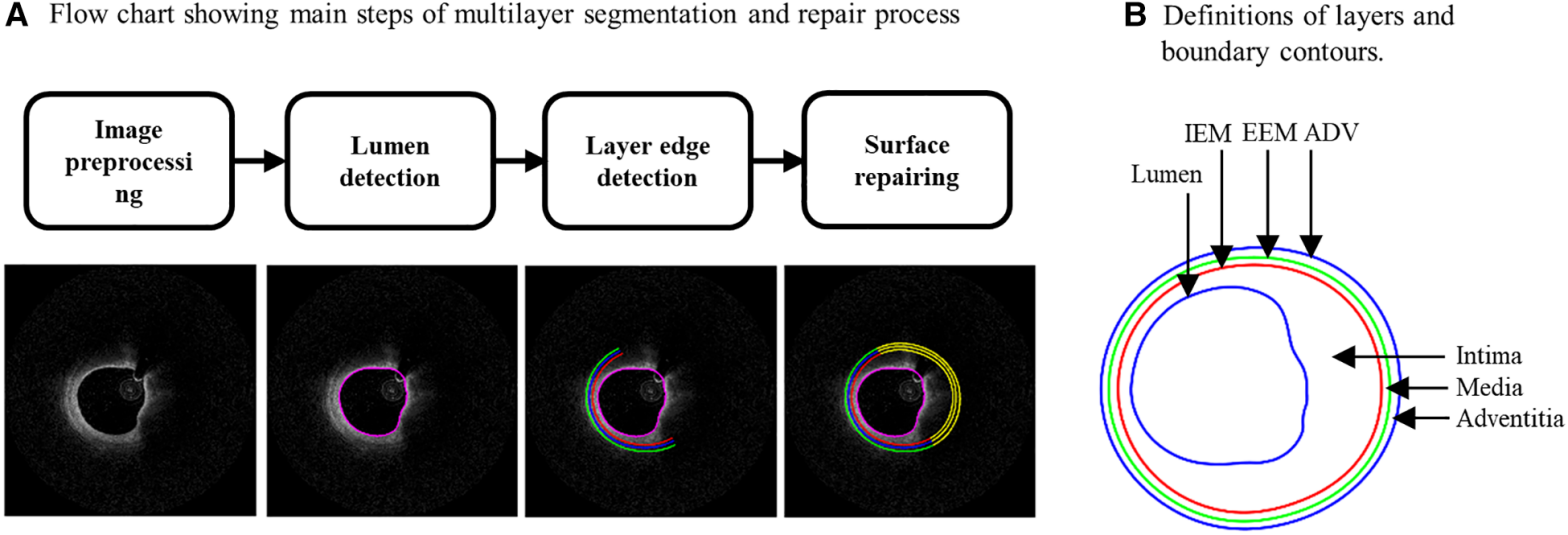
(**A**) Flow chart showing main steps of an in-house automatic multi-layer segmentation and repair process; (**B**) a sample slice showing segmented three layer contours. IEM, Internal elastic membrane; EEM, External elastic membrane; ADV, adventitia-periadventitia interface.

**Figure 2:**
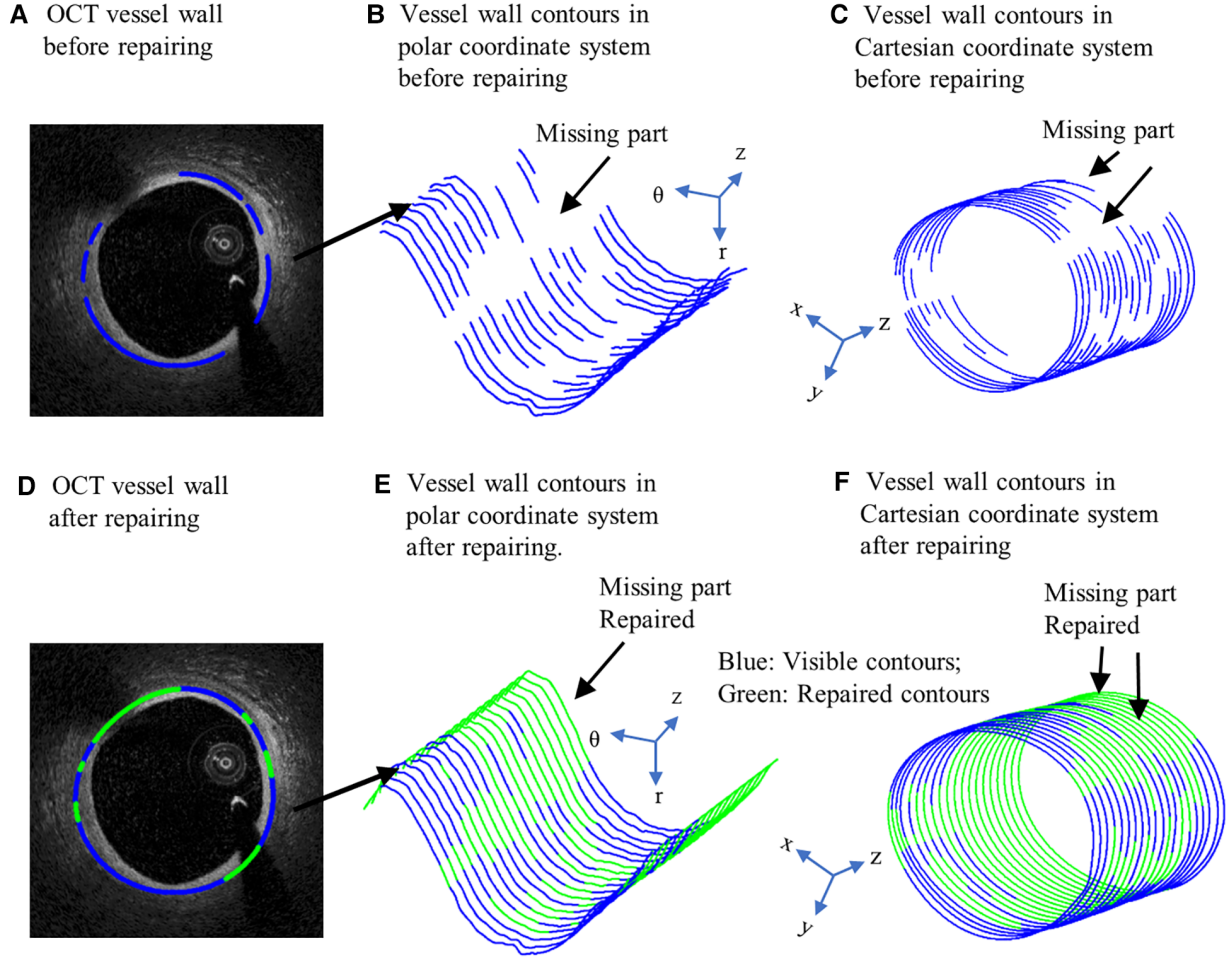
Schematic of OCT image repairing. (**A**) OCT vessel wall before repairing. (**B**) Vessel wall contours in polar coordinate system before repairing. (**C**) Vessel wall contours in Cartesian coordinate system before repairing. (**D**) OCT vessel wall after repairing. (**E**) Vessel wall contours in polar coordinate system after repairing. (**F**) Vessel wall contours in Cartesian coordinate system after repairing. Blue contours: Visible contours; Green contours: Repaired contours for missing parts.

It is well-known that arteries have a three-layer structure: intima, media, and adventitia. While most publications used single-layer models, multi-layer models are desirable for more realistic modeling of the artery and more accurate plaque stress/strain calculations. For this purpose, multi-layer automatic segmentation of OCT images was performed to get vessel layer structures using a MATLAB-based method (MATLAB R2021a, MathWorks, USA) previously introduced by Huang et al. (9). Figure 1 presents a flow chart of this segmentation process alongside a sample slice illustrating the three layers segmented from the OCT image. Figure 2 provided more details for the repair process. The OCT image was segmented by our automatic segmentation program which processes OCT images in polar coordinate system. We first applied edge detection algorithms, specifically a modified Canny method, to segment the visible portions (Figures 2A–C). Subsequently, cubic spline surface fitting was employed to fit the surface function *r* = *r*(z, *θ*) to get the locations of missing portions (Figures 2D–F). For accuracy validation, the automated segmentation results were compared with manual segmentations and good agreement were found. The plaque samples used in this study were mostly of circular shape which made the interpolation easier. The segmented slices with contours for the intima, media, and adventitia layers were then employed for model construction.

### 2.2 Multi-layer models with residual stress inclusion and layer-specific material properties

Plaque stress/strain conditions play an important role in plaque progression, remodeling and potential rupture. Accurate models are the base for reliable and precise stress/strain calculations. We recently introduced an OCT multi-layer segmentation method and OCT-based multi-layer plaque models (10). In this paper, we are adding residual stress to multi-layer models for further improvement. For comparison purpose, single-layer models with and without residual stress inclusion were also constructed to show differences for both single-layer and multi-layer models. The following four models were constructed for each patient: (a) multi-layer model without residual stress; (b) single-layer model without residual stress; (c) multi-layer model with residual stress inclusion; and (d) single-layer model with residual stress inclusion. Our novel multi-layer model with residual stress inclusion added layer structure and residual stress to the other three models (one or both) and should provide more realistic representation of the physical artery/plaque among the four models. Details for material properties and residual modeling process are given below.

#### 2.2.1 Layer-specific material models and material parameters

**Figure 3:**
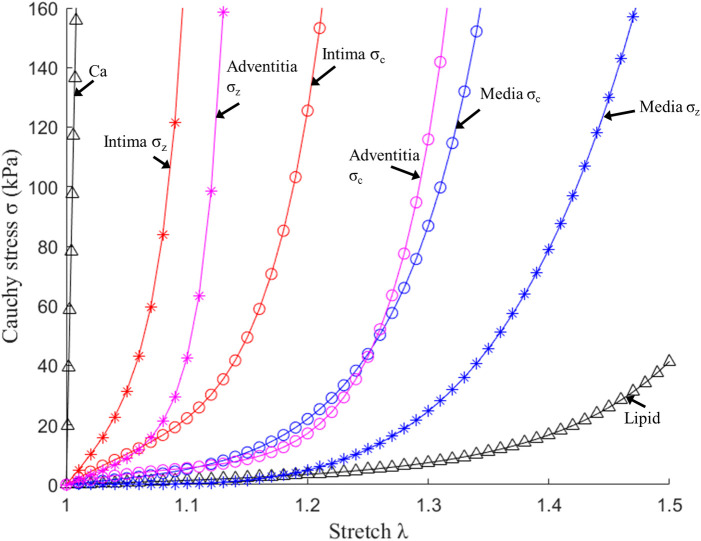
Stress–stretch curves of three layers and plaque components derived from the mooney-rivlin models and used in finite element modelling. *σ*_c_, circumferential stress; *σ*_z_, axial stress (10).

Vessel material properties for the three layers (and the single-layer) were assumed to be hyperelastic, anisotropic, nearly incompressible, and homogeneous, while plaque components like lipid and calcifications were considered isotropic. The modified Mooney–Rivlin material models were used for the layers using parameter values from available literature (18, 19). The modified Mooney-Rivlin material models were used because that were available on ADINA (Adina R & D, Watertown, MA, USA) which was used to solve our finite element models. We did model comparisons for Mooney-Rivlin model, Fung-type model and Choi-Vito model using biaxial testing data (4 coronary and 5 carotid plaque samples) and the modified Mooney-Rivlin material models provided better fitting accuracies (19). The strain energy functions are represented by Equations 1 and 2 given below.

(1) $$\begin{array}{c} W_{iso}=c_{1}(I_{1}-3)+c_{2}(I_{2}-3)+D_{1}[exp(D_{2}(I_{1}-3))-1] \end{array}$$

(2) $$\begin{array}{c} W_{aniso}=W_{iso}+\frac{K_{1}}{K_{2}}\{exp[K_{2}{\left(I_{4}-1\right)}^{2}]-1\} \end{array}$$

where $I_{1}=\sum (C_{\mathrm{ii}})$, $I_{2}=\frac{1}{2}[I_{1}^{2}-C_{\mathrm{ij}}C_{\mathrm{ij}}]$, *I*_1_ and *I*_2_ denote the first and second invariants of right Cauchy–Green deformation tensor $C=[C_{\mathrm{ij}}]=F^{T}F$, $F=[F_{\mathrm{ij}}]=[\partial x_{i}/\partial a_{j}]$;$\left(x_{i}\right)$ is current position; $\left(a_{j}\right)$ s original position; $I_{4}=\lambda_{\theta }^{2}cos^{2}\varphi +\lambda_{z}^{2}\mathrm{si}n^{2}\varphi$, where $\lambda_{\theta }$, $\lambda_{z}$, are the principal stretches associated with circumferential and axial direction and φ is the angle between the fiber reinforcement and the circumferential direction in individual layers. *c*_1_, *c*_2_, *D*_1_, *D*_2_, *K*_1_ and *K*_2_ are material parameters. Parameter values for the vessel layers and p*l*aque components used in our models are (10, 18): Intima: *c*_1 _= −169.23 kPa, *c*_2 _= 177.40 kPa, *D*_1 _= 2.4 kPa, *D*_2 _= 13, *K*_1 _= 32 kPa, *K*_2 _= 36; Media: *c*_1 _= −67.25 kPa, *c*_2 _= 35.01 kPa, *D*_1 _= 17 kPa, *D*_2 _= 2, *K*_1 _= 7 kPa, *K*_2 _= 4, φ = 24.9°; Adventitia; *c*_1 _= −94.44 kPa, *c*_2 _= 102.42 kPa, *D*_1 _= 0.8 kPa, *D*_2 _= 10, *K*_1 _= 10 kPa, *K*_2 _= 40, φ = 75.3°; lipid core: *c*_1 _= 0.5 kPa, *c*_2 _= 0 kPa, *D*_1 _= 0.5 kPa, *D*_2 _= 1.5; calcification: *c*_1 _= 920 kPa, *c*_2 _= 0 kPa, *D*_1 _= 360 kPa, and *D*_2 _= 2.0. For single-layer models, intima parameter values were used for the entire vessel wall. Figure 3 depicts the stress–stretch curves of three layers derived from the modified Mooney–Rivlin material models.

#### 2.2.2 Multi-layer 3D thin-slice model with residual stress inclusion

**Figure 4:**
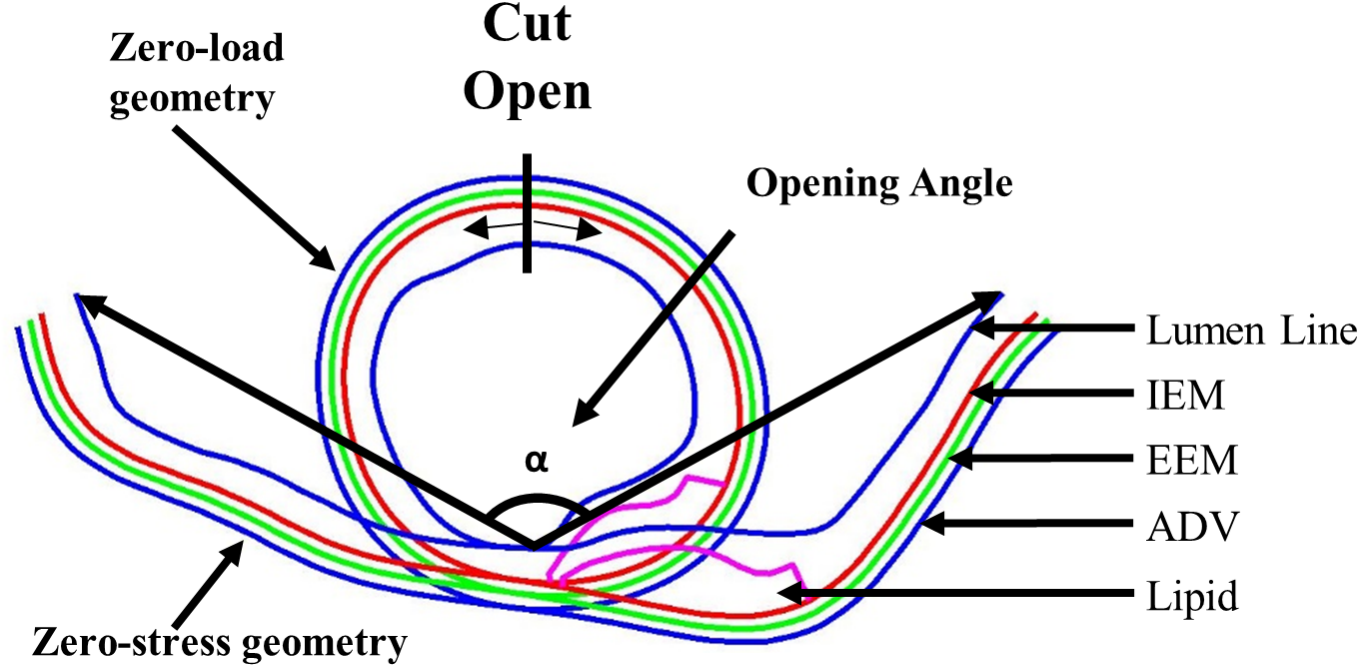
Vessel zero-load and zero-stress geometries with three-layer structure and opening angle.

*In vivo* OCT image data were obtained when the blood vessel was under pressure and axially stretched. So the vessel image data should be shrunk circumferentially (radially) and axially to obtain its no-load state. The no-load geometry needs to be cut open to release the residual stress to obtain its stress-free state (11). To construct accurate coronary plaque models, it is imperative to initiate from zero-stress state, which is a condition not readily extractable from medical image. To obtain vessel zero-stress state from its *in vivo* state, the vessel was shrunk circumferentially (radially) and axially to achieve a zero-load state first. A 5% axial pre-shrink and the circumferential pre-shrink process were used to get vessel no-load state (16). The circumferential shrinkage rate was set as 5% initially and adjusted by an iterative procedure until the pressurized slice under diastolic pressure matched the *in vivo* OCT slice (tolerance <0.1%). Subsequently, the zero-load geometry was cut open to release the residual stress to obtain its zero-stress state. The opening-up process adhered to two fundamental assumptions: (1) vessel wall volume was conserved and (2) the circumference of the middle line of the vessel wall remained unchanged. The opening angle of the vessel sector was postulated to be 120°, which was derived from the average of eight human coronary artery samples (19). Figure 4 illustrates the vessel's transition from zero-load to zero-stress state. The zero-stress geometry (cut-open slice) was then used as the starting geometry for model construction.

### 2.3 Three-step model solution procedure

Three-dimensional (3D) thin-slice models were constructed for 10 OCT slices from 10 patients using multi-layer segmented data obtained from our programs. Four types of models (multi-layer with and without residual stress, single-layer with and without residual stress) were constructed for each patient, resulting in 40 thin-slice models in total. The 3D thin-slice model was made by adding a 0.5 mm thickness to each slice to better approximate full 3D models, yet maintaining the low construction cost about the same as that of 2D models. A three-step modeling procedure was used in the modeling process starting from zero-stress state to recover the vessel *in vivo* state: (a) wrap the zero-stress vessel slice to its no-load geometry (note now the closed vessel slice carries residual stress/strain as desired). The wrapping process is accomplished by applying prescribed displacement to the two cut-openings of the opened vessel slice and bringing them to come together (contact); (b) stretch the vessel axially to its length *in vivo*; and (c) pressurize the vessel to recover *in vivo* geometry (16). Finite element mesh was generated using a commercial finite-element package ADINA 9.6 (Adina R & D, Watertown, MA, USA). Given the complex morphologies of plaques, a “volume-fitting” technique was employed to divide the 3-D plaque, intima, media and adventitia domains into many small “volumes” to curve-fit the irregular vessel geometry with plaque component inclusions (20). This technique was crucial for achieving convergent plaque finite element models. Mesh analysis was performed by decreasing mesh size by 10% (in each dimension) incrementally until solution differences were less than 2%. The optimized mesh was then chosen for our simulations. The thin-slice models were solved following our established procedures (21). Because stress/strain are tensors, maximum principal stress and maximum principal strain (called stress and strain from here on, respectively) were chosen as their scale representatives for stress/strain comparisons.

### 2.4 Data extraction and analysis

**Figure 5:**
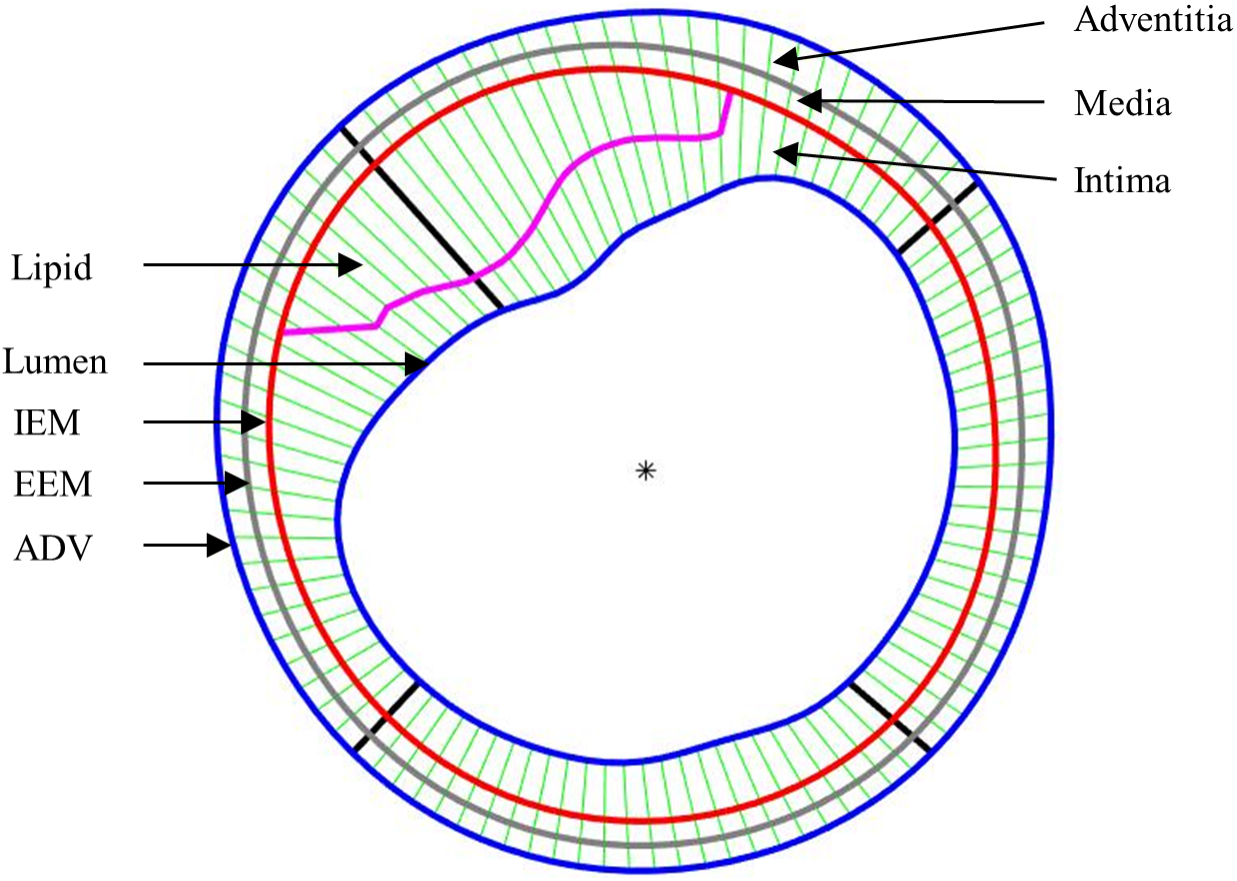
Schematic plot demonstrating the piecewise equal-step method to select 100 points for data extraction.

After the models were solved, stress and strain values from 100 data points for each slice at plaque inner wall (called plaque stress/strain for simplicity) and out-wall (out-wall stress/strain) were extracted to compare results and investigate the impact of residual stress on plaque stress/strain calculations. The ratios (percentage) of maximum and mean stress/strain values between models with and without residual stress were calculated to investigate the impact of residual stress inclusion on stress/strain calculations. Since plaque slices often have irregular and nonuniform wall thickness, a four-quarter method was introduced to connect lumen points and out-wall points to avoid data distortion by thicker plaques (22). Figure 5 gives an illustration of the four-quarter method and the three layers of the vessel: intima, media and adventitia. The boundary between intima and media is called internal elastic membrane (IEM). The boundary between media and adventitia is called external elastic membrane (EEM). The boundary between adventitia and other peripheral tissues is called adventia-periadventitia interface (ADV).

## 3 Results

The following comparisons of plaque stress/strain values from multi-layer models (M-Model) and single-layer models (S-Model) with and without residual stress inclusion were made to investigate the impact of residual stress on plaque stress/strain conditions: (a) plaque stress/strain values on inner wall from multi-layer models with and without residual stress; (b) plaque stress/strain values on inner wall from single-layer models with and without residual stress; (c) plaque stress/strain values on out-wall from multi-layer models with and without residual stress; (d) plaque stress/strain values on out-wall from single-layer models with and without residual stress.

Stress/strain results from our novel multi-layer models with residual stress should be the most accurate among the 4 models compared, while single-layer model comparison results are also of interest since single-layer models are used in most publications. Plaque research has been focused on inner wall plaque stress/strain conditions. However, results from outer walls were reported since they were all important factors in plaque progression and remodeling process (8).

### 3.1 Multi-layer model with residual stress inclusion reduced inner wall maximum and mean plaque stresses by 38.57% and 59.70%

Table 2 gave maximum and mean plaque stress values on vessel inner wall from multi-layer and single-layer models and comparisons for models with and without residual stress for the 10 patients studied. From multi-layer models with residual stress inclusion, the inner wall maximum and mean plaque stress values (averaged over 10 patients) were 148.53 kPa and 50.68 kPa respectively, which were 38.57% and 59.70% lower than the values from corresponding models without residual stress. From single-layer models with residual stress, the inner wall maximum and mean plaque stress values were 59.14 kPa and 6.89 kPa, which were 61.46% and 94.72% lower than the values from corresponding models without residual stress. The influence of residual stress on plaque stress exhibited large patient variations for both multi-layer and single-layer models. It is evident that residual stress has large impact on plaque stress calculations.

**Table 2:** Inner wall maximum and mean plaque stress comparisons between models with and without residual stress.

Plaque	Maximum plaque stress (kPa)				Mean plaque stress (kPa)			
	M-Model with residual stress	M-Model no residual stress	S-Model with residual stress	S-Model no residual stress	M-Model with residual stress	M-Model no residual stress	S-Model with residual stress	S-Model no residual stress
P1	124.74	238.71	57.96	146.95	42.89	109.61	1.29	70.81
	52.26%	100.00%	39.44%	100.00%	39.13%	100.00%	1.83%	100.00%
P2	60.54	126.87	6.34	65.94	26.52	71.94	−3.85	47.59
	47.72%	100.00%	9.62%	100.00%	36.87%	100.00%	−8.09%	100.00%
P3	176.22	195.62	72.97	143.34	33.19	99.16	−11.56	65.54
	90.08%	100.00%	50.91%	100.00%	33.47%	100.00%	−17.65%	100.00%
P4	178.66	346.11	29.35	214.84	58.69	159.02	3.29	85.64
	51.62%	100.00%	13.66%	100.00%	36.91%	100.00%	3.84%	100.00%
P5	101.76	249.30	67.26	129.92	37.29	120.55	4.02	79.93
	40.82%	100.00%	51.77%	100.00%	30.93%	100.00%	5.03%	100.00%
P6	293.74	260.33	96.29	141.88	111.33	138.60	37.03	94.36
	112.83%	100.00%	67.87%	100.00%	80.33%	100.00%	39.24%	100.00%
P7	289.90	388.15	158.10	204.86	107.47	203.26	34.75	124.70
	74.69%	100.00%	77.17%	100.00%	52.87%	100.00%	27.87%	100.00%
P8	109.26	334.76	49.61	209.43	44.93	99.93	11.85	71.71
	32.64%	100.00%	23.69%	100.00%	44.96%	100.00%	16.53%	100.00%
P9	47.06	124.67	45.79	103.49	−0.54	38.72	−3.25	34.47
	37.75%	100.00%	44.24%	100.00%	−1.40%	100.00%	−9.44%	100.00%
P10	103.46	140.02	7.80	111.01	45.06	92.10	−4.69	73.13
	73.88%	100.00%	7.03%	100.00%	48.92%	100.00%	−6.41%	100.00%
Mean ± STD	148.53 ± 86.35	240.45 ± 94.72	59.14 ± 44.74	147.16 ± 49.37	50.68 ± 34.62	113.29 ± 45.94	6.89 ± 16.52	74.79 ± 24.71
Mean ± STD (%)	61.43% ± 25.77%	100.00%	38.54% ± 24.46%	100.00%	40.30% ± 20.42%	100.00%	5.28% ± 17.83%	100.00%

M-Model, multi-layer model; S-Model, single-layer model.

### 3.2 Multi-layer model maximum and mean plaque strains on vessel inner wall were reduced by 31.96% and 52.84% with residual stress inclusion

Similar to Table 2, Table 3 gave maximum and mean plaque strain values on vessel inner wall and comparisons from multi-layer and single-layer models with and without residual stress inclusion. From multi-layer models with residual stress inclusion, the inner wall maximum and mean plaque strain values (average for 10 patients) were 0.158 and 0.065 respectively, which were 31.96% and 52.84% lower than the values from corresponding models without residual stress. From single-layer models with residual stress, the inner wall maximum and mean plaque strain values were 0.134 and 0.049, which were 34.27% and 56.68% lower than the values from corresponding models without residual stress. Overall, plaque strain comparison results and patient variations were similar to plaque stress behaviors.

**Table 3:** Inner wall maximum and mean plaque strain comparisons between models with and without residual stress.

Plaque	Maximum plaque strain				Mean plaque strain			
	M-Model with residual stress	M-Model no residual stress	S-Model with residual stress	S-Model no residual stress	M-Model with residual stress	M-Model no residual stress	S-Model with residual stress	S-Model no residual stress
P1	0.194	0.308	0.130	0.282	0.062	0.161	0.024	0.140
	63.00%	100.00%	46.06%	100.00%	38.40%	100.00%	17.29%	100.00%
P2	0.097	0.256	0.094	0.242	0.032	0.137	0.029	0.119
	38.06%	100.00%	39.02%	100.00%	23.02%	100.00%	24.14%	100.00%
P3	0.155	0.214	0.186	0.177	0.069	0.119	0.061	0.099
	72.44%	100.00%	105.39%	100.00%	57.77%	100.00%	62.21%	100.00%
P4	0.141	0.353	0.108	0.284	0.079	0.146	0.052	0.112
	39.85%	100.00%	37.98%	100.00%	54.10%	100.00%	46.85%	100.00%
P5	0.204	0.197	0.164	0.170	0.066	0.128	0.059	0.106
	103.89%	100.00%	96.49%	100.00%	51.71%	100.00%	55.30%	100.00%
P6	0.199	0.287	0.149	0.269	0.090	0.144	0.061	0.123
	69.40%	100.00%	55.38%	100.00%	62.41%	100.00%	49.85%	100.00%
P7	0.203	0.217	0.171	0.197	0.102	0.158	0.067	0.130
	93.39%	100.00%	86.81%	100.00%	64.87%	100.00%	51.23%	100.00%
P8	0.118	0.180	0.074	0.155	0.064	0.117	0.053	0.100
	65.60%	100.00%	47.63%	100.00%	54.53%	100.00%	52.33%	100.00%
P9	0.148	0.189	0.214	0.184	0.034	0.109	0.067	0.105
	78.58%	100.00%	116.52%	100.00%	31.20%	100.00%	64.11%	100.00%
P10	0.125	0.222	0.054	0.207	0.053	0.156	0.014	0.144
	56.20%	100.00%	25.99%	100.00%	33.63%	100.00%	9.86%	100.00%
Mean ± STD	0.158 ± 0.039	0.242 ± 0.057	0.134 ± 0.052	0.217 ± 0.049	0.065 ± 0.022	0.137 ± 0.019	0.049 ± 0.019	0.118 ± 0.016
Mean ± STD (%)	68.04% ± 20.88%	100.00%	65.73% ± 32.36%	100.00%	47.16% ± 14.45%	100.00%	43.32% ± 19.13%	100.00%

M-Model, multi-layer model; S-Model, single-layer model.

**Figure 6:**
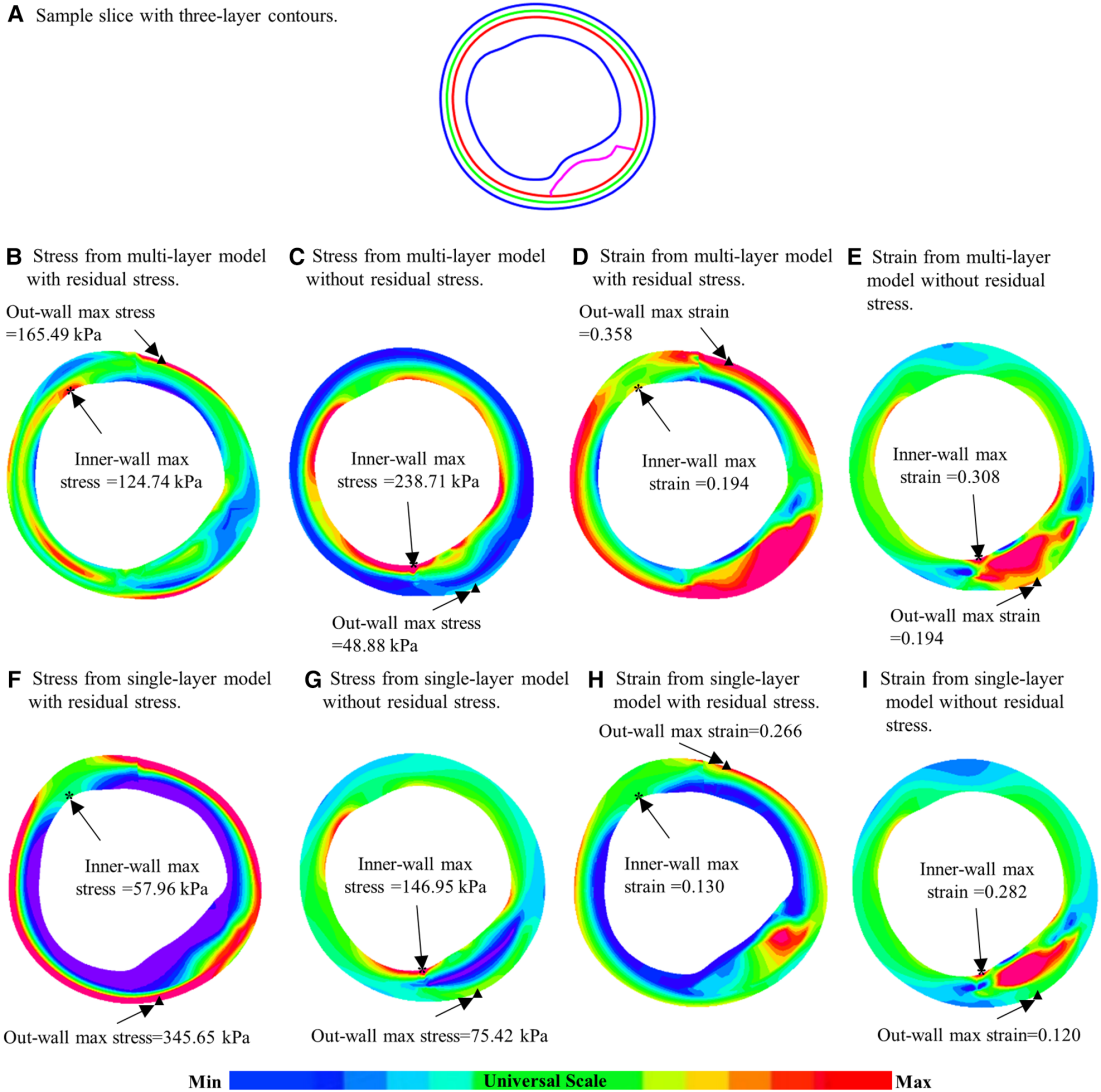
Comparison of plaque stress/strain distributions of four models using a sample slice. (**A**) A sample slice with three-layer contours. (**B–E**) Stress plots from multi-layer models with and without residual stress inclusion; (**F**–**I**) Stress from single-layer models with and without residual stress inclusion. *: Maximum stress/strain on inner wall; ▴: maximum stress/strain on out-wall.

To demonstrate model differences more clearly, Figure 6 gave plaque stress/strain distribution plots from the four models using a sample slice. Figures 6B–E showed stress and strain plots from multi-layer models while Figures 6F–I showed single-layer model stress/strain plots. Models with residual stress inclusion had lower maximum stress/strain values on inner wall and higher maximum stress/strain values on out-wall compared to models without residual stress.

### 3.3 Out-wall maximum and mean out-wall stresses increased by 572.84% and 432.03% for multi-layer models with residual stress inclusion

Plaque stress and strain on vessel out-wall play an important role in vessel remodeling process (8). Table 4 gave maximum and mean out-wall stress values from multi-layer and single-layer models and comparisons for models with and without residual stress for the 10 patients studied. From multi-layer models with residual stress, the maximum and mean out-wall stress values were 211.15 kPa and 97.03 kPa, which were 572.84% and 432.03% higher than the values from corresponding models without residual stress. From single-layer models with residual stress, the maximum and mean out-wall stress values were 400.28 kPa and 194.55 kPa, which were 770.93% and 591.31% higher than the values from corresponding models without residual stress.

**Table 4:** Out-wall maximum and mean out-wall stress comparisons between models with and without residual stress.

Plaque	Maximum out-wall stress (kPa)				Mean out-wall stress (kPa)			
	M-Model with residual stress	M-Model no residual stress	S-Model with residual stress	S-Model no residual stress	M-Model with residual stress	M-Model no residual stress	S-Model with residual stress	S-Model no residual stress
P1	165.49	48.88	345.65	75.42	89.30	24.85	185.90	41.01
	338.56%	100.00%	458.33%	100.00%	359.34%	100.00%	453.29%	100.00%
P2	209.17	24.49	305.38	36.51	99.72	16.79	183.28	24.90
	854.16%	100.00%	836.33%	100.00%	594.04%	100.00%	736.21%	100.00%
P3	246.51	25.66	358.05	42.23	96.99	16.58	163.43	25.38
	960.49%	100.00%	847.82%	100.00%	584.91%	100.00%	643.83%	100.00%
P4	231.92	46.17	271.38	72.96	102.69	29.81	149.97	48.19
	502.30%	100.00%	371.95%	100.00%	344.51%	100.00%	311.21%	100.00%
P5	114.51	35.07	516.57	64.67	60.00	20.68	220.26	35.55
	326.54%	100.00%	798.82%	100.00%	290.13%	100.00%	619.59%	100.00%
P6	199.11	44.76	294.56	80.32	84.22	22.83	154.52	38.71
	444.89%	100.00%	366.73%	100.00%	368.89%	100.00%	399.20%	100.00%
P7	233.05	46.68	364.80	90.51	91.82	29.16	168.47	53.02
	499.21%	100.00%	403.04%	100.00%	314.89%	100.00%	317.76%	100.00%
P8	164.42	32.12	265.36	57.63	80.48	19.80	146.60	32.70
	511.84%	100.00%	460.45%	100.00%	406.48%	100.00%	448.32%	100.00%
P9	391.54	24.99	780.87	28.08	153.02	10.90	327.86	15.32
	1,567.07%	100.00%	2,781.11%	100.00%	1,403.62%	100.00%	2,140.55%	100.00%
P10	155.81	21.54	500.19	36.12	112.05	17.15	245.26	29.09
	723.35%	100.00%	1,384.68%	100.00%	653.45%	100.00%	843.18%	100.00%
Mean ± STD	211.15 ± 75.66	35.04 ± 10.73	400.28 ± 159.65	58.45 ± 21.63	97.03 ± 24.26	20.85 ± 5.94	194.55 ± 56.51	34.39 ± 11.39
Mean ± STD (%)	672.84% ± 378.09%	100.00%	870.93% ± 743.90%	100.00%	532.03% ± 332.34%	100.00%	691.31% ± 539.32%	100.00%

M-Model, multi-layer model; S-Model, single-layer model.

### 3.4 Multi-layer models with residual stress inclusion increased maximum and mean out-wall strain by 240.21% and 299.79% respectively

Table 5 gave maximum and mean out-wall strain values from multi-layer and single-layer models and comparisons for models with and without residual stress for the 10 patients studied. From multi-layer models with residual stress inclusion, the maximum and mean out-wall strain values were 0.369 and 0.225, which were 240.21% and 299.79% higher than those from corresponding models without residual stress. From single-layer models with residual stress inclusion, the maximum and mean out-wall strain values were 0.265 and 0.164, 235.51% and 264.88% higher than those from corresponding models without residual stress, respectively.

**Table 5:** Out-wall maximum and mean out-wall strain comparisons between models with and without residual stress.

Plaque	Maximum out-wall strain				Mean out-wall strain			
	M-Model with residual stress	M-Model no residual stress	S-Model with residual stress	S-Model no residual stress	M-Model with residual stress	M-Model no residual stress	S-Model with residual stress	S-Model no residual stress
P1	0.358	0.194	0.266	0.120	0.246	0.087	0.176	0.072
	185.02%	100.00%	221.29%	100.00%	284.02%	100.00%	244.89%	100.00%
P2	0.389	0.099	0.275	0.078	0.248	0.049	0.189	0.039
	394.26%	100.00%	353.28%	100.00%	501.91%	100.00%	479.25%	100.00%
P3	0.396	0.073	0.258	0.059	0.227	0.055	0.161	0.051
	545.80%	100.00%	439.17%	100.00%	411.36%	100.00%	312.73%	100.00%
P4	0.397	0.148	0.279	0.083	0.238	0.082	0.151	0.058
	267.60%	100.00%	334.96%	100.00%	288.94%	100.00%	260.47%	100.00%
P5	0.435	0.097	0.303	0.081	0.171	0.065	0.114	0.056
	450.40%	100.00%	374.63%	100.00%	262.11%	100.00%	205.59%	100.00%
P6	0.326	0.130	0.229	0.099	0.200	0.070	0.154	0.061
	250.78%	100.00%	231.07%	100.00%	286.78%	100.00%	251.55%	100.00%
P7	0.306	0.141	0.218	0.109	0.205	0.086	0.152	0.067
	216.83%	100.00%	200.96%	100.00%	237.90%	100.00%	227.53%	100.00%
P8	0.307	0.098	0.213	0.073	0.194	0.062	0.146	0.055
	313.53%	100.00%	293.67%	100.00%	313.06%	100.00%	266.24%	100.00%
P9	0.451	0.110	0.371	0.065	0.245	0.026	0.209	0.020
	409.14%	100.00%	572.17%	100.00%	943.74%	100.00%	1,038.05%	100.00%
P10	0.326	0.088	0.244	0.073	0.271	0.058	0.193	0.053
	368.79%	100.00%	333.85%	100.00%	468.03%	100.00%	362.55%	100.00%
Mean ± STD	0.369 ± 0.052	0.118 ± 0.036	0.265 ± 0.047	0.084 ± 0.020	0.225 ± 0.031	0.064 ± 0.019	0.164 ± 0.028	0.053 ± 0.015
Mean ± STD (%)	340.21% ± 113.62%	100.00%	335.51% ± 111.76%	100.00%	399.79% ± 211.41%	100.00%	364.88% ± 249.68%	100.00%

M-Model, multi-layer model; S-Model, single-layer model.

## 4 Discussion

### 4.1 The impact of residual stress inclusion on stress/strain calculations

The importance of plaque stress/strain calculations for vulnerable plaque progression and rupture risk assessment is well recognized. To our knowledge, this paper should be a first report for impact of residual stress on plaque stress/strain calculations using patient-specific multi-layer models based on segmented 3-layer OCT data. Our findings revealed that residual stress inclusion reduced inner wall maximum and mean plaque stresses by 38.57% and 59.70% and increased out-wall maximum and mean plaque stresses by 572.84% and 432.03%. Using multi-layer model with residual stress inclusion as the base, the multi-layer model without residual stress inclusion over-estimated inner wall maximum and mean stress by 61.8% and 123.5%, respectively. The single-layer model situation is even more scary: the single layer model without residual stress inclusion over-estimated inner wall maximum and mean stress by 148.8% and 985.5% (using values from single layer model with residual stress), respectively. The incorporation of residual stress significantly impacts stress and strain calculations, which has great potential for enhancing the prediction accuracy of plaque progression and vulnerability. Furthermore, it might provide doctors with better patient screening strategies, enabling the timely application of appropriate interventions or conservative therapies. This will optimize the process of patient management, diagnosis, and treatment of cardiovascular diseases, ultimately aiming to improve patient outcomes in cardiovascular healthcare. There is also a well-accepted threshold stress value 300 kPa for plaques with high vulnerability (23, 24). When interpreting model results, the associated model assumptions should be taken into consideration.

Multi-layer model residual stress inclusion also increased out-wall maximum and mean plaque stresses by 572.84% and 432.03%. That led to a more uniform distribution of stress within the vessel wall (see Figure 5), which is consistent with the principle that the human body would regulate vessel stress to be uniform to achieve optimal functionality (8, 11). Essentially, residual stress introduced compressive circumferential stress in the intima and circumferential “stretch” in the adventitia. Interestingly, negative mean plaque stress values at inner wall location were observed in 1 multi-layer model and 4 single-layer models with residual stress inclusion in our study.

### 4.2 Comparison of multi-layer and single layer models with residual stress inclusion

Multi-layer models exhibited more uniform stress distributions compared to single-layer models. In multi-layer models with residual stress, the average mean stresses of 10 plaques at inner and out-wall locations were 50.68 kPa and 97.03 kPa, respectively. While from single-layer models with residual stress, the inner wall and out-wall stress values were 6.89 kPa and 194.55 kPa. Multi-layer models with and without residual stress inclusion had mean inner wall stresses 50.68 kPa and 113.29 kPa (averaged over 10 patients), respectively, compared to 6.89 kPa and 74.79 kPa from single-layer models. For out-wall mean stress values, multi-layer models with and without residual stress inclusion had 97.03 kPa and 20.85 kPa, compared to 194.55 kPa and 34.39 kPa from single-layer models. Stress differences from single-layer models with and without residual stress inclusion had much greater differences. Layer-specific material properties are the cause of these large differences.

### 4.3 Potential clinical benefits of multi-layer models with residual stress inclusion

Multi-layer models with residual stress inclusion could lead to more accurate plaque stress and strain calculations which could have a wide range of clinical applications including plaque vulnerability assessment, prediction of plaque progression and vulnerability change through diverse biomechanical indicators, as well as exploring the correlation between mechanical conditions and the incidence of future major adverse cardiovascular events. Plaque cap stress (which is on inner layer of the vessel) is well-recognized risk factor for possible plaque rupture.

Table 2 gave maximum and mean plaque stress values on vessel inner wall from multi-layer and single-layer models and comparisons for models with and without residual stress for the 10 patients studied. From multi-layer models with residual stress inclusion, the inner wall maximum and mean plaque stress values (averaged over 10 patients) were 148.53 kPa and 50.68 kPa respectively, which were 38.57% and 59.70% lower than the values from corresponding models without residual stress. From single-layer models with residual stress, the inner wall maximum and mean plaque stress values were 59.14 kPa and 6.89 kPa, which were 61.46% and 94.72% lower than the values from corresponding models without residual stress. The average of the inner-layer maximum stress of the 10 patients from the multi-layer models with residual stress inclusion was 148.53 ± 86.35 kPa while the value from the multilayer models without residual stress was 240.45 ± 94.72, a 61.8% over-estimate. Another potential use of the multi-layer models with residual stress was for artery remodeling which could be closed related to vessel out-wall stress/strain conditions. The average of the outer-layer maximum stress of the 10 patients from the multi-layer models with residual stress inclusion was 211.15 ± 75.66 kPa, while the value from the multilayer models without residual stress was 35.04 ± 10.73 kPa, only 16.6% of the value from models with residual stress. Those results suggest that residual stress inclusion would have considerable impact for vulnerable plaque and artery remodeling investigations.

While we demonstrated the considerable impact of residual stress on stress/strain calculations and subsequent clinical applications, it should be noted that clinical acceptance and implementation remain to be big challenges. Our small data size is a serious limitation. Large scale studies are needed to get solid validation of our initial results and then final clinical acceptance. Another challenge is model construction cost. The open-close process in the construction of models with residual stress was done manually which was very labor intensive. Automation of the modeling process is a must for potential clinical implementations.

### 4.4 The use of 3D thin-slice models vs. full 3D models

3D thin-slice models were used in this study with two major reasons: (a) 3D thin-slice model could provide better approximation than what 2D model would since it did have slice thickness and axial pre-shrink-stretch was performed to take axial residual stress into consideration; (b) model construction time for 3D thin-slice model was only slightly more than 2D models, but much less compared to full 3D models. The ultimate goal of computational modeling and vulnerable plaque research is to implement the modeling and mechanical analysis for possible clinical applications and providing stress/strain conditions to aid vulnerable plaque detection, cardiovascular disease diagnosis, management and possible prevention of drastic events such as heart attack and/or stroke. 3D models are too far away from actual implementation due to their labor cost. 3D thin-slice models could be a compromise in between: reasonable accuracy and labor cost which makes practical implementation possible. Wang Q et al. provided multi-patient model comparisons and reported that the errors from 3D thin-slice models were around 10% compared to full 3D models (25). That was encouraging.

### 4.5 Limitations and future directions

We would like to acknowledge some limitations of our study: (a) Small patient size is a severe limitation. This is a pilot study to investigate the impact of residual stress on stress/strain calculations using a small patient size. Model construction with multilayers and residual stress inclusion is very time consuming due to the manual open-close process. Larger-scale studies are needed to further validate the impact of residual stress, and to explore its impact across various patient groups, in terms of age, sex, different plaque types and comorbidities, etc. This will enable a more comprehensive understanding of residual stress's role in plaque dynamics; (b) 3D thin-slice models were used to reduce labor cost. Techniques should be developed to construct full 3D models with residual stress inclusion to improve accuracy of stress/strain calculations, but also keeping labor cost at acceptable level; (c) Due to lack of available vessel residual stress data and material properties, an average opening angle and material parameter values from the literature were utilized in this study. It should be noted that it is not possible to obtain patient-specific opening angle from live patients. It is also challenging to obtain patient-specific multi-layer vessel material parameters.

## 5 Conclusion

In this study, multi-layer and single-layer coronary plaque models with and without residual stress inclusion were constructed for 10 patients based on automatically segmented three-layer OCT images to quantify the impact of residual stress on stress/strain calculations. Our results showed that residual stress plays a critical role in the stress distribution of vessel tissues, and led to reduced inner wall plaque stress and increased out-wall stress. Larger scale studies are needed to further improve model accuracy and validate our findings.

## Data Availability

The original contributions presented in the study and data supporting the conclusions are included in the article. Further inquiries can be directed to the corresponding author Dalin Tang, dtang@wpi.edu.

## References

[1] Ku DN Giddens DP Zarins CK Glagov S. Pulsatile flow and atherosclerosis in the human carotid bifurcation. Positive correlation between plaque location and low oscillating shear stress. Arteriosclerosis. (1985) 5:293–302. 10.1161/01.atv.5.3.293 3994585

[2] Bluestein D Alemu Y Avrahami I Gharib M Dumont K Ricotta JJ Influence of microcalcifications on vulnerable plaque mechanics using FSI modeling. J Biomech. (2008) 41(5):1111–8. 10.1016/j.jbiomech.2007.11.029 18258240

[3] Samady H Eshtehardi P Mcdaniel MC Suo J Dhawan SS Maynard C Coronary artery wall shear stress is associated with progression and transformation of atherosclerotic plaque and arterial remodeling in patients with coronary artery disease. Circulation. (2011) 124(7):779–88. 10.1161/CIRCULATIONAHA.111.021824 21788584

[4] Tang D Teng Z Canton G Yang C Ferguson M Huang X Sites of rupture in human atherosclerotic carotid plaques are associated with high structural stresses: an in vivo MRI-based 3D fluid-structure interaction study. Stroke. (2009) 40(10):3258–63. 10.1161/STROKEAHA.109.558676 19628799 PMC2753753

[5] Wentzel JJ Corti R Fayad ZA Wisdom P Macaluso F Winkelman MO Does shear stress modulate both plaque progression and regression in the thoracic aorta? Human study using serial magnetic resonance imaging. J Am Coll Cardiol. (2005) 45(6):846–54. 10.1016/j.jacc.2004.12.026 15766817

[6] Costopoulos C Maehara A Huang Y Brown AJ Gillard JH Teng Z Heterogeneity of plaque structural stress is increased in plaques leading to mace: insights from the prospect study. JACC Cardiovasc Imaging. (2020) 13(5):1206–18. 10.1016/j.jcmg.2019.05.024 31326476 PMC7198978

[7] Fung YC Liu SQ. Strain distribution in small blood vessels with zero-stress state taken into consideration. Am J Physiol. (1992) 262(2 Pt 2):H544–52. 10.1152/ajpheart.1992.262.2.H544 1539714

[8] Rachev A Taylor WR Vito RP. Calculation of the outcomes of remodeling of arteries subjected to sustained hypertension using a 3D two-layered model. Ann Biomed Eng. (2013) 41(7):1539–53. 10.1007/s10439-012-0727-9 23296999

[9] Huang M Maehara A Tang D Zhu J Wang L Lv R Human coronary plaque optical coherence tomography image repairing, multilayer segmentation and impact on plaque stress/strain calculations. J Funct Biomater. (2022) 13(4):213–26. 10.3390/jfb13040213 36412854 PMC9680523

[10] Huang M Maehara A Tang D Zhu J Wang L Lv R Comparison of multilayer and single-layer coronary plaque models on stress/strain calculations based on optical coherence tomography images. Front Physiol. (2023) 14:1251401. 10.3389/fphys.2023.1251401 37608838 PMC10440539

[11] Fung YC. A First Course in Continuum Mechanics. Englewood Cliffs: New Jersey: Prentice Hall (1994).

[12] Holzapfel GA Sommer G Auer M Regitnig P Ogden RW. Layer-specific 3D residual deformations of human aortas with non-atherosclerotic intimal thickening. Ann Biomed Eng. (2007) 35(4):530–45. 10.1007/s10439-006-9252-z 17285364

[13] Holzapfel GA Ogden RW. Modelling the layer-specific three-dimensional residual stresses in arteries, with an application to the human aorta. J R Soc Interface. (2010) 7(46):787–99. 10.1098/rsif.2009.0357 19828496 PMC2874228

[14] Delfino A Stergiopulos N Moore JE Meister JJ. Residual strain effects on the stress field in a thick wall finite element model of the human carotid bifurcation. J Biomech. (1997) 30(8):777–86. 10.1016/S0021-9290(97)00025-0 9239562

[15] Ohayon J Dubreuil O Tracqui P Le Floc’h S Rioufol G Chalabreysse L Influence of residual stress/strain on the biomechanical stability of vulnerable coronary plaques: potential impact for evaluating the risk of plaque rupture. Am J Physiol Heart Circ Physiol. (2007) 293(3):H1987–96. 10.1152/ajpheart.00018.2007 17604326

[16] Wang L Zhu J Samady H Monoly D Zheng J Guo X Effects of residual stress, axial stretch, and circumferential shrinkage on coronary plaque stress and strain calculations: a modeling study using IVUS-based near-idealized geometries. J Biomech Eng. (2017) 139(1):014501. 10.1115/1.4034867 PMC5125309 27814429

[17] Pierce DM Fastl TE Rodriguez-Vila B Verbrugghe P Fourneau I Maleux G A method for incorporating three-dimensional residual stretches/stresses into patient-specific finite element simulations of arteries. J Mech Behav Biomed Mater. (2015) 47:147–64. 10.1016/j.jmbbm.2015.03.024 25931035

[18] Holzapfel GA Sommer G Gasser CT Regitnig P. Determination of layer-specific mechanical properties of human coronary arteries with nonatherosclerotic intimal thickening and related constitutive modeling. Am J Physiol Heart Circ Physiol. (2005) 289(5):H2048–58. 10.1152/ajpheart.00934.2004 16006541

[19] Kural MH Cai M Tang D Gwyther T Zheng J Billiar KL Planar biaxial characterization of diseased human coronary and carotid arteries for computational modeling. J Biomech. (2012) 45(5):790–8. 10.1016/j.jbiomech.2011.11.019 22236530 PMC3294096

[20] Yang C Bach RG Zheng J Naqa IE Woodard PK Teng Z In vivo IVUS-based 3-D fluid-structure interaction models with cyclic bending and anisotropic vessel properties for human atherosclerotic coronary plaque mechanical analysis. IEEE Trans Biomed Eng. (2009) 56(10):2420–8. 10.1109/TBME.2009.2025658 19567341 PMC2918807

[21] Lv R Maehara A Matsumura M Wang L Zhang C Huang M Using optical coherence tomography and intravascular ultrasound imaging to quantify coronary plaque cap stress/strain and progression: a follow-up study using 3D thin-layer models. Front Bioeng Biotechnol. (2021) 9:713525. 10.3389/fbioe.2021.713525 34497800 PMC8419245

[22] Wang Q Tang D Wang L Canton G Wu Z Hatsukami TS Combining morphological and biomechanical factors for optimal carotid plaque progression prediction: an MRI-based follow-up study using 3D thin-layer models. Int J Cardiol. (2019) 293:266–71. 10.1016/j.ijcard.2019.07.005 31301863 PMC6710108

[23] Finet G Ohayon J Rioufol G. Biomechanical interaction between cap thickness, lipid core composition and blood pressure in vulnerable coronary plaque: impact on stability or instability. Coron Artery Dis. (2004) 15(1):13–20. 10.1097/00019501-200402000-00003 15201616

[24] Cardoso L Weinbaum S. Changing views of the biomechanics of vulnerable plaque rupture, a review. Ann Biomed Eng. (2014) 42(2):415–31. 10.1007/s10439-013-0855-x 23842694 PMC3888649

[25] Wang Q Tang D Wang L Meahara A Molony D Samady H Multi-patient study for coronary vulnerable plaque model comparisons: 2D/3D and fluid–structure interaction simulations. Biomech Model Mechanobiol. (2021) 20(4):1383–97. 10.1007/s10237-021-01450-8 33759037 PMC8298251

